# RIPOSTE: a framework for improving the design and analysis of laboratory-based research

**DOI:** 10.7554/eLife.05519

**Published:** 2015-05-07

**Authors:** Nicholas GD Masca, Elizabeth MA Hensor, Victoria R Cornelius, Francesca M Buffa, Helen M Marriott, James M Eales, Michael P Messenger, Amy E Anderson, Chris Boot, Catey Bunce, Robert D Goldin, Jessica Harris, Rod F Hinchliffe, Hiba Junaid, Shaun Kingston, Carmen Martin-Ruiz, Christopher P Nelson, Janet Peacock, Paul T Seed, Bethany Shinkins, Karl J Staples, Jamie Toombs, Adam KA Wright, M Dawn Teare

**Affiliations:** Cardiovascular Biomedical Research Unit, University of Leicester, Leicester, United Kingdom; Leeds Institute of Rheumatic and Musculoskeletal Medicine, University of Leeds, Leeds, United Kingdom; Leeds Institute of Rheumatic and Musculoskeletal Medicine, NIHR Leeds Musculoskeletal Biomedical Research Unit, Leeds, United Kingdom; Department of Primary Care and Public Health Sciences, King's College London, London, United Kingdom; Applied Computational Genomics, University of Oxford, Oxford, United Kingdom; Department of Infection and Immunity, University of Sheffield, Sheffield, United Kingdom; The Florey Institute, University of Sheffield, Sheffield, United Kingdom; Department of Cardiovascular Sciences, University of Leicester, Leicester, United Kingdom; NIHR Diagnostic Evidence Co-Operative Leeds, Leeds Teaching Hospitals NHS Trust, Leeds, United Kingdom; Musculoskeletal Research Group, Institute of Cellular Medicine, University of Newcastle, Newcastle, United Kingdom; Newcastle Hospitals NHS Trust, Newcastle, United Kingdom; NIHR Biomedical Research Centre at Moorfields Eye Hospital NHS Foundation Trust and UCL Institute of Ophthalmology, London, United Kingdom; London School of Hygiene and Tropical Medicine, London, United Kingdom; Centre for Pathology, Imperial College, London, United Kingdom; Clinical Trials and Evaluation Unit, School of Clinical Sciences, University of Bristol, Bristol, United Kingdom; Department of Paediatric Haematology, Sheffield Children's NHS Foundation Trust, Sheffield, United Kingdom; Royal London Hospital, London, United Kingdom; Respiratory Biomedical Research Unit, Royal Brompton and Harefield NHS Trust, London, United Kingdom; Institute for Ageing and Health, Newcastle University, Newcastle, United Kingdom; Department of Cardiovascular Sciences, NIHR Leicester Cardiovascular Biomedical Research Unit, University of Leicester, Leicester, United Kingdom; Division of Health and Social Care Research, Kings College London, London, United Kingdom; NIHR Biomedical Research Centre at Guy's and St Thomas' NHS Foundation, London, United Kingdom; Division of Women's Health, King's College London, London, United Kingdom; Nuffield Department of Primary Care Health Sciences, University of Oxford, Oxford, United Kingdom; Clinical and Experimental Sciences, University of Southampton and NIHR Southampton Respiratory Biomedical Research Unit, Southampton General Hospital, Southampton, United Kingdom; Department of Molecular Neuroscience, Institute of Neurology, University College London, London, United Kingdom; Institute of Lung Health, Respiratory Biomedical Unit, University Hospitals of Leicester NHS Trust, Leicester, United Kingdom; Sheffield School of Health and Related Research, University of Sheffield, Sheffield, United Kingdom; McGill University, Canada

**Keywords:** experimental design, statistical design, reproducibility, interdisciplinary research, science forum, none

## Abstract

Lack of reproducibility is an ongoing problem in some areas of the biomedical sciences. Poor experimental design and a failure to engage with experienced statisticians at key stages in the design and analysis of experiments are two factors that contribute to this problem. The RIPOSTE (Reducing IrreProducibility in labOratory STudiEs) framework has been developed to support early and regular discussions between scientists and statisticians in order to improve the design, conduct and analysis of laboratory studies and, therefore, to reduce irreproducibility. This framework is intended for use during the early stages of a research project, when specific questions or hypotheses are proposed. The essential points within the framework are explained and illustrated using three examples (a medical equipment test, a macrophage study and a gene expression study). Sound study design minimises the possibility of bias being introduced into experiments and leads to higher quality research with more reproducible results.

**DOI:** http://dx.doi.org/10.7554/eLife.05519.001

## Introduction

Laboratory-based studies play a central role in preclinical biomedical research, encompassing a diverse range of techniques and spanning a broad range of fields across the biomedical sciences. For example, investigations of the biological pathways underpinning drug response or microbial pathogenesis, the assessment of safety and efficacy of interventions, and the discovery of biomarkers all rely on laboratory-based methods for at least some stages of observation, measurement and/or processing. Despite this key role, approaches to the design, analysis and reporting of laboratory studies can be highly varied. Moreover, the frequently dynamic nature of laboratory based research can mean that studies are often complex and may consist of various exploratory components, which may not be fully documented when results are published. This can lead to a lack of transparency about the research methodology, and may prevent any results and findings from being successfully reproduced.

Lack of reproducibility (or ‘irreproducibility’) is an acknowledged problem within biomedicine that has recently been gaining increased attention (Begley and Ellis, 2012; Begley, 2013; The Economist, 2013; Macleod et al., 2014). Attempts to independently confirm or follow-up on spurious research findings waste time, money (which may have public or charitable origins) and resources, and also raises ethical concerns. Initiatives aiming to address irreproducibility in the biomedical sciences are therefore underway (Errington et al., 2014; Morrison, 2014). Initial efforts have largely focussed on improving reporting standards from research publications. For example, both Science and Nature have recently introduced new reporting guidelines that aim to improve the transparency of research disseminated in their journals (Nature, 2013; McNutt, 2014). Attempts to harmonise and improve reporting standards across particular types of study have also been made. The Minimum Information About a Microarray Experiment (MIAME) (Brazma, 2009) initiative targets experimental protocols in microarray experiments and other ‘omics’ studies, while the REMARK guidelines (Altman et al., 2012) focus on the appropriate and transparent use of statistical methods in tumour marker prognostic studies. A recent report from the Institute of Medicine in the US also focuses on ‘omics’ studies (Institute of Medicine, 2012). Several other relevant guidelines for reporting health related research can be found through the EQUATOR network (equator-network.org). A common theme in these guidelines is the appropriate and transparent reporting of statistical methods.

Whilst the above initiatives aim to improve transparency in published laboratory based research, a focus only on the reporting of studies does not address other key factors that may also contribute to irreproducibility. For instance, a recent retraction of a MIAME compliant study (Sebastiani et al., 2011) demonstrates that targeting reporting standards alone cannot prevent irreproducibility. A large number of other retractions have also been highlighted (www.retractionwatch.com), increasing the focus on what contributes to the problem and how to tackle it (Irizarry et al., 2005; Baggerly and Coombes, 2009; Prinz et al., 2011; Sebastiani et al., 2011; Begley and Ellis, 2012; Lambert and Black, 2012; Parker and Leek, 2012; Freedman and Inglese, 2014).

Several factors may lead to irreproducibility in laboratory studies. As highlighted above, poor reporting can limit the ability to accurately reproduce results. Although general methodology and procedures are usually reported, key details needed to guarantee that an entire analysis pipeline can be reproduced are often missing, such as information about a study's methods and/or analysis. This may include information about the modality of data handling and manipulation, version of software and/or libraries used, and implementation of the statistical methods.

Issues relating to the generation of data, including study design and methods to minimise the introduction of bias, may also contribute to irreproducibility. Any bias introduced into a study often cannot be removed and may impact on the results in ways that may be difficult to quantify (Bogardus et al., 1999). These issues may stem from practices within the laboratory itself; for example, unwanted variation posed by batch effects or other confounding variables can systematically and irreversibly distort the measurements taken within a study unless appropriately accounted for at the design stage. Technical issues relating to the analysis may also lead to errors; for instance, incorrectly distinguishing between repeated and independent measurements can increase the likelihood of obtaining false positive or false negative results.

A lack of formal guidance on the process of laboratory study design may also give rise to irreproducibility. Although in some respects laboratory-based research is highly regulated, such regulation largely relates to materials, processes and ethics rather than focussing on aspects of study design or improving methodological rigor. For example, procedures and protocols must be approved by the Control of Substances Hazardous to Health Regulations (COSHH), while pre-clinical pharmaceutical and medical device research is governed very strictly by Good Laboratory Practice (GLP) regulations. Clinical studies using human samples are subject to ethical review, research governance and the International Committee on Harmonisation of Good Clinical Practice (ICH GCP) and may also be subject to the Human Tissue Act (2004). Certain laboratory work is also conducted under accreditation from UKAS and CPA or to ISO/BSI standards. In contrast to other methods of experimental research such as clinical trials, however, none of these regulations specifically addresses study design and there is often no formal requirement to produce a study-specific protocol or analysis plan in advance of data collection.

The existing culture where novel research is rewarded over and above attempts to replicate findings may also contribute to irreproducibility. Those who attempt to replicate results currently face expenses in terms of time and resources, and can find it hard to publish their findings whether they confirm or not (Ioannidis, 2005, 2006). This may contribute to the well-known phenomenon of ‘publication bias’, where positive but potentially one-off or chance novel results disproportionately enter the literature at the expense of negative findings. Failing to adequately document negative findings can also lead to publication bias, and may lead to others unnecessarily repeating the work in future. Bias towards publication of statistically significant results has been shown to be substantively greater for observational and laboratory-based studies than for randomised clinical trials (Easterbrook et al., 1991).

It is now generally accepted that poor study design is a major problem in laboratory based research (Collins and Tabak, 2014). While most scientists will have received training in experimental design in an abstract form, it may be difficult to put it into practice, especially when some experiments can be conducted by a single researcher. Currently this poor design is being picked up at the reporting stage as was the case in clinical research some 30 years ago. For instance, in the 1980s weaknesses in the reporting of clinical studies led to a number of initiatives to improve statistical awareness and understanding. As a result, reporting guidelines were developed (Moher et al., 2001; Schulz et al., 2010) to promote the reporting of key methodological components and results that enable study bias to be assessed and to support evidence synthesis. This recognition of the key role of statistical principles in study design and analysis has resulted in integrated and critical involvement by statisticians in all aspects of clinical trials. Ethical concerns have led regulatory bodies to impose strict standards concerning all aspects of the design, analysis and reporting of clinical trials, which ensure that they are properly planned and implemented.

A clinical trial can be considered the equivalent of a single experiment to test a specific hypothesis. These single experiment trials require their own funding and generally result in at least two publications; the protocol and the results on completion. By contrast in basic science it is very unusual for the results of a single experiment to be published in isolation. It is more common to find a series of experiments presented, linked with inductive and deductive reasoning. This tendency to present a broad range of linked experiments and results in a single publication has been a barrier to the development of appropriate reporting guidelines. Some journals are now actively promoting the submission of short follow-on reports (the *Research Advance* in eLife) or breakthrough articles where simple but important questions are addressed (Corey et al., 2014). Although the methods employed in laboratory studies are diverse and experiments can be completed within very short time frames, much can be learnt from the standards upheld in trials. Trials are designed and managed by regular consultation within full, multidisciplinary teams. Such teams can involve health-economists, computer scientists and statisticians, as well as clinicians, scientists and/or qualitative researchers. Input from the full interdisciplinary team at all stages of a study helps to ensure that trials are optimally designed, making efficient use of resources and avoiding potential difficulties at the analysis stage. Trials are long term projects where protocols are first established, participants are recruited and then endpoints are measured. By contrast, in laboratory research many experiments may be conducted in parallel at many levels within a research group, and the rigid clinical trial design structure would not allow the flexibility required for new research to emerge. We assert that greater consideration of the principles of good experimental design coupled with early and regular discussion amongst all the members of the research team will help improve the design, analysis and reporting of laboratory based studies. This, in turn, should lead to higher quality data and reproducible research. Such improvements will require a gear change from all involved in the field especially from research funders.

To support the implementation of such an integrated approach we have developed the RIPOSTE framework, which draws together key elements of laboratory study design and analysis that may contribute to reproducibility. The framework is accompanied by three hypothetical case-studies to demonstrate the discussion that may follow the consideration of each prompt point. The overall aim of the framework is to support discussion within a multidisciplinary research team (including the statistician), to ensure that potential sources of bias and/or variability have been considered and, where possible, eliminated at the design stage. We are aware that scientific advances can be made through a mix of inductive and deductive reasoning. This framework is focussed to support more discussion in the deductive stages when hypotheses and specific questions are proposed.

The framework was developed in two stages. The NIHR Statistics Group held a laboratory research studies day, during which the initial project was conceived and major elements for the framework identified. A prompt-list using items commonly encountered in reporting guidelines was then constructed and revised to be relevant for laboratory experiments at the design stage. For the second stage we invited 12 statisticians and 12 laboratory scientists to a one-day workshop where the framework was piloted as a means to facilitate discussion on aspects of study design and analysis. The framework was trialled in small groups: two scientists and two statisticians worked in each group and the framework was tested using examples supplied by the scientists. At the end of the workshop feedback was obtained and suggested modifications to the framework were collated. Modifications were made and further feedback was obtained from the RIPOSTE consortium via an online survey. In the survey delegates were asked to score the inclusion of items on a 0–10 scale (high score to retain item). Items receiving a median score less than 8 were removed, and any which had been scored 0 by at least one respondent, irrespective of the median score, were revised if necessary in line with the respondents' comments. Suggestions on the structure and presentation were also incorporated.

We present here a framework to support early discussions within a multidisciplinary research team, which should consist of both scientists and statisticians. The framework contains a comprehensive list of the details that facilitate reproduction of research and is intended to promote discussion about key aspects of the design, conduct and analysis of a planned laboratory study. The framework offers a series of prompts that raise pertinent questions to facilitate shared understanding of the research and the environment in which it is being undertaken.

The catch-all term ‘laboratory studies’ covers a wide range of study types (Box 4), and some aspects of the framework will not always be applicable in all studies. Similarly, some aspects of the framework will not always be relevant for discussion with statisticians, but nevertheless concern issues that still require careful consideration within the research team. We see this framework as a useful toolbox in the hands of the scientist, which takes and builds upon many points raised in recent journal and topic specific publication guidelines. Our workshop confirmed that it can take a long time for a statistician to fully understand the basic designs of a series of experiments when first presented. This is often due to lack of familiarity with the field of application. We felt that using some carefully selected case-studies to demonstrate how the prompts in the framework can be used would help both statisticians and scientists in its implementation. We have, therefore, included three hypothetical case studies as examples which have been selected to cover a broad spectrum of biomedical laboratory settings. The first (Box 1) is a study of combinations of components of automated medical dosing equipment, where the motivation is to look for equivalent performance. The second study (Box 2) examines macrophage activity when cells are infected with bacteria and treated with a drug. This experiment illustrates replicate measurement, treatment and infection control contrasts and plate or batch effects. The third (Box 3) is a gene expression study in patients with hypertension (cases) and without hypertension (controls), where the aim is to identify genes that are differentially expressed. This example allows us to illustrate multiple hypothesis testing and a variety of sources of batch effects from tissue processing through to RNA analysis. The framework is presented in Table 1; this sets out the major prompts for topics to be considered and gives some brief notes for each. The following sections follow the headings in Table 1 and provide a more detailed breakdown and discussion of items from the framework, clarifying our recommendations.

10.7554/eLife.05519.003

Box 1. **Example: Elastomer pump study**

A study is planned to assess a new type of disposable elastomer pump and catheter for use in delivering anaesthetic directly to wounds following major surgery. The study aims to assess whether the new pump and catheter—or combinations of the new pump and catheter with an existing pump and catheter—achieve an acceptable flow rate over time (i.e., where an ‘acceptable’ flow rate is defined as within 15% of the set rate). The researchers also wish to assess whether the performance of the equipment declines after with reuse.

Methods and materials: The experimental set-up is presented in Box 1 Figure 1. In order to mimic clinical practice, the flow rate will be set to 4 ml/hr, and each experiment will run over a period of 48 hr. Automated weight measurements of the pump will be taken every 2 hr via a laptop, and concurrent measurements of the room temperature will also be made as temperature may impact upon the flow. Each type of pump (P = existing pump; p = new pump) is to be tested with each type of catheter (C = existing catheter; c = new catheter). Four experiments will be run simultaneously over four units, with each experiment repeated three times before changing equipment (i.e., each experiment will be run *in triplicate*). Due to limited study resources, only four pumps and four catheters of each type are available for use.

**Box 1 Figure 1.**
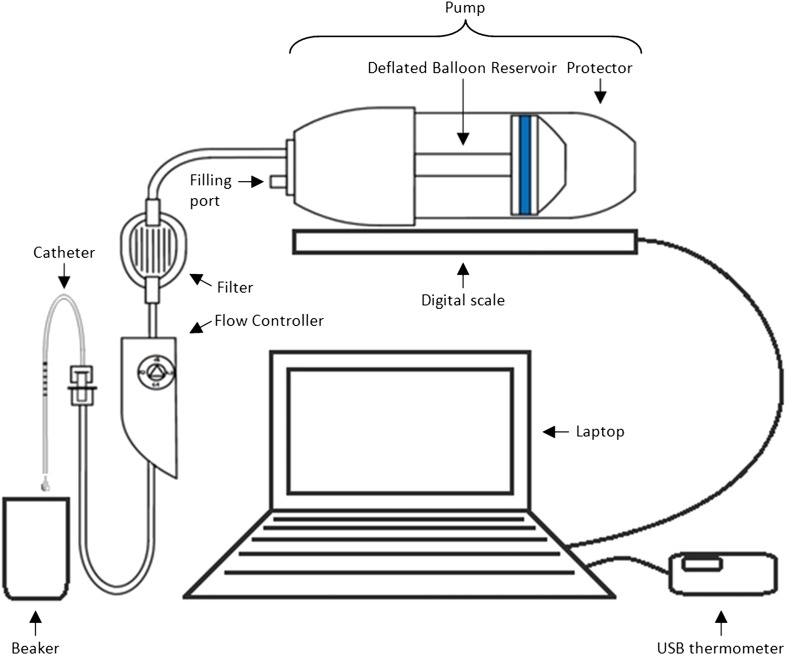
Equipment set-up for elastomer pump experiment. **DOI:** http://dx.doi.org/10.7554/eLife.05519.005

Design: Box 1 Table 1 illustrates two possible arrangements of the pump/catheter combinations over the 4 units. Design A runs a particular combination over all 4 units at the same time before switching to the next combination, while Design B tests the four different combinations of pumps and catheters simultaneously before alternating the order of the combinations over the units after each set of triplicate experiments.

**Box 1 Table 1.** Two potential study designs in which either (a) four pumps and four catheters of the same type are tested simultaneously or (b) pump and catheter types are balanced during each 48 hr period of data collection, assuming only four pump-catheter units can be used concurrently and each is tested for 48 hr, three times in succession **DOI:** http://dx.doi.org/10.7554/eLife.05519.004

Arrangement	Duration	Bench 1	Bench 2	Bench 3	Bench 4
Suboptimal design with potential for confounding					
1	48 hrs × 3	**P C**	**P C**	**P C**	**P C**
2	48 hrs × 3	**P** c	**P** c	**P** c	**P** c
3	48 hrs × 3	p **C**	p **C**	p **C**	p **C**
4	48 hrs × 3	p c	p c	p c	p c
Optimal, balanced design					
1	48 hrs × 3	**P C**	**P** c	p **C**	p c
2	48 hrs × 3	**P** c	**P C**	**p** c	p **C**
3	48 hrs × 3	p c	p **C**	**P** c	**P C**
4	48 hrs × 3	p **C**	p c	**P C**	**P** c

There are four benches of equipment being tested, each with one of each type of pump and one of each type of catheter (**P** = existing pump; p = new pump, **C** = existing catheter; c = new catheter).

**DOI:** http://dx.doi.org/10.7554/eLife.05519.003

10.7554/eLife.05519.006

Box 2. **Example: Macrophage Study**

A series of experiments are planned to characterise macrophage activity (cytokine production and apoptosis) when cells which are infected with bacteria are treated with a drug. Blood will be taken from multiple volunteer donors to obtain peripheral blood mononuclear cells from which differentiated macrophages are produced. The macrophages will be infected with a specific dose of bacteria and treated with a drug. The cytokine production and apoptosis will be measured at intervals over 24 hr. The panel of 10 cytokines will be measured by a multiplex bead system. Each donor will be processed with internal controls so the four combinations of infection status (infected/mock infected) and treatment (drug treatment/control) will be measured.

Research Question: Does treatment with a specific drug to cells infected with bacteria affect macrophage immune function measured by cytokine production and apoptosis?

The basic experimental design will include:

1. The assessment of baseline cytokine production in infected and mock infected macrophages.
2. The time course of cytokine production following the infection point, captured by measuring levels every 2 hr.
3. The matched design ensures that cells from each donor can be studied for response to both infection and treatment. Exactly half of the infected and half of the mock infected macrophages are treated with the drug and this is balanced over all donors.
4. The four combinations of treatment and infection will be processed in parallel on the samples.

Box 2 Figure 2 illustrates two possible ways that macrophages from just two donors might be arranged, for incubation in a single experiment on two eight well sections of a plate. Each subject has macrophages grown in eight wells, four of these will be infected with the same bacteria, and four will be mock infection controls. Two of the infected and two of the mock infected will be treated with the drug. Hence for each donor the measurement of variables under each condition is done twice (i.e., in duplicate). Arrangements A and B show a total of four possible plate arrangements. Some arrangements have conditions or donors clustered or organised into rows or columns. The two plates for ‘A’ make it easy for the infectious agent to be dosed out in one block, whereas ‘B’ has all the wells to be treated with the drug in a single column. In three of the plates, wells from different donors are never direct neighbours; however, the infection is done in blocks or pairs of neighbours. The diagram shown here shows only a part of a larger plate. Plate sizes of 24 or 96 well plates are available for use here; therefore multiple plates need to be used. The bead system for measuring cytokine levels uses assays which are automated, however, the assessment of apoptosis involves visual inspection and counting of cells. The colour of the medium indicates exactly which samples are infected and which are treated, which means the measurements cannot be taken ‘blind’ to the treatment.

**Box 2 Figure 1.**
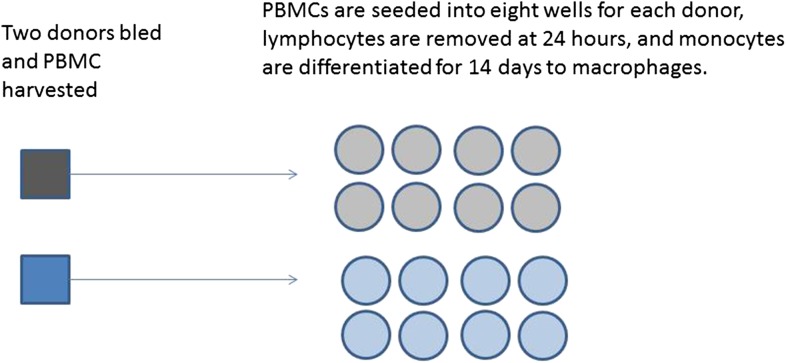
Production of differentiated macrophages from donor samples. **DOI:** http://dx.doi.org/10.7554/eLife.05519.007

**Box 2 Figure 2.**
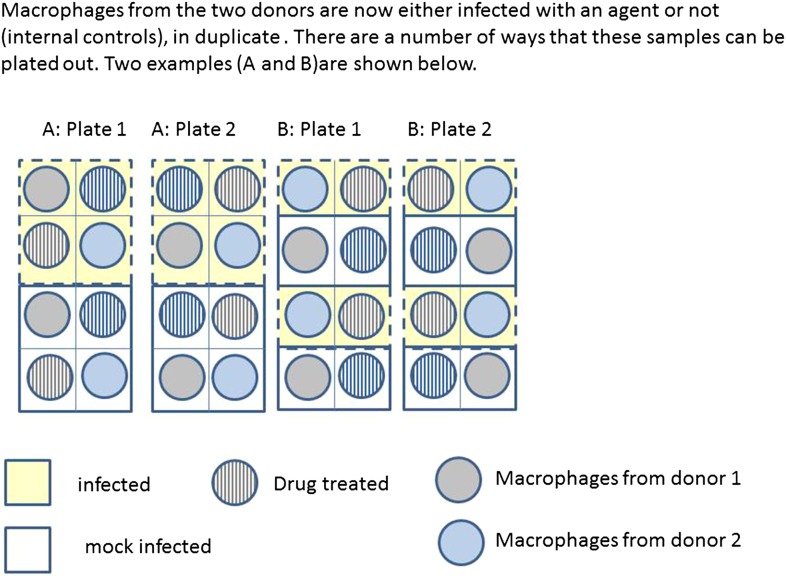
Infection and treatment of donor macrophages. **DOI:** http://dx.doi.org/10.7554/eLife.05519.008

**DOI:** http://dx.doi.org/10.7554/eLife.05519.006

10.7554/eLife.05519.009

Box 3. **Example: Gene expression study using RNA-seq**

A study is designed to examine differences in gene expression in kidney tissue taken from human subjects who exhibit a hypertensive phenotype and those who do not. Gene expression will be assessed using RNA sequencing (RNA-seq), which quantifies the expression of both genes and the RNA transcripts produced by genes. Each gene can have multiple transcripts—in humans there are approximately 213,000 known transcripts produced by ∼62,000 genes.

Aims of the study: To identify genes that are differentially expressed in hypertensive patients compared to normotensive controls. This study will function as a discovery stage to pick up differentially expressed genes to take forward for evaluation in a larger sample.

Research Question/Hypotheses: The aim will be to identify transcripts and genes that differ in expression between cases and controls. A hypothesis will be tested for each transcript to assess whether or not it associates with the disease status. A transcript would be declared as differentially expressed if the log_2_ fold difference between cases and controls is statistically significant after accounting for multiple testing using the false-discovery rate.

Outcomes of interest: The primary outcome is the expression level for each transcript or gene; there will be multiple of these (10,000 s). The measurement of the outcome will involve three stages. First the kidney tissue samples are collected and the RNA extracted and assessed for quality using the RNA Integrity Number (RIN), secondly samples are then to be sent to a bioscience company for sequencing. Finally the sequence data is received from the company and a toolkit such as Tuxedo will be used for data processing. There is the potential to report on the use of standard protocols in each of these steps.

Materials and Techniques: There are SOPs for the RNA extraction and the methods employed within the bioscience company. The material will need to be run in batches, so a mix (random or balanced) of cases and controls will be sent in each batch and each batch will contain at least one common sample to assist in the control of batch effects. The quality of the RNA (as it arrives at the company) will be a predictor of the quality of the sequencing. The sample processing and source of the samples (i.e., the preparation before sending for sequencing) may mean that there are systematic (batch) differences between cases and controls.

Software: Specialist software exists for each stage of this planned analysis. The Tuxedo suite is designed to process the raw data output from the sequencing. PEER has been developed to identify and correct for sources of variation. The statistical analysis will be done using R Bioconductor. A workflow diagram to indicate how the options for each program were set at each stage of the data processing and analysis will be constructed during the study and will be used at the analysis and reporting stage.

Constraints: The main constraint is the cost of the sequencing, hence the preference is to opt for fewer subject samples so sequencing can be done at a higher coverage. The maximum number of samples to be processed is around 40.

Randomisation and Blinding: There is no treatment to be applied. However cases and controls will be randomly mixed in batches for shipping to the sequencing company. The bioscience company will be blind to the case control status.

Statistical Analysis: There are two groups, cases and controls, all analyses will adjust for the confounders age, sex and body mass index. The Limma package in R Bioconductor fits linear models to each gene/transcript, then ‘normalises’ across genes and estimates p-values using an empirical Bayes estimator. The multiple testing will be accounted for with the FDR correction. The correction will be for the full number of transcripts analysed (i.e., post all ‘Quality control’ (QC) criteria). Sequencing uncertainty is reflected in low expression values so genes with uncertain reads are likely to not meet the threshold. The QC requirements are that a transcript must be expressed in a minimum number of samples to be included for further analysis.

Validation: To ensure the results are not due to a technical artefact the most significant results will be validated using a different technology (the same samples run through a different technique).

**DOI:** http://dx.doi.org/10.7554/eLife.05519.009

10.7554/eLife.05519.010

Box 4. **Examples of laboratory studies**

What do we mean by ‘laboratory study’?

- A study in which any aspect of the procedure or analysis is carried out in a research facility/lab.
- May be in vivo (e.g., imaging) or in vitro (e.g., cell culture).
- Includes both experimental and observational studies, but excludes interventional trials^*^.
- May involve estimation, hypothesis generation or hypothesis testing/confirmation.
- Can be small (e.g., within a single lab) or large scale (e.g., multi-centre genome-wide association studies).

*Specific guidance is available for interventional trials, however many of the RIPOSTE recommendations will be relevant

**DOI:** http://dx.doi.org/10.7554/eLife.05519.010

**Table 1.** RIPOSTE discussion framework **DOI:** http://dx.doi.org/10.7554/eLife.05519.002

Item	Prompt/Consideration	Details (relevance of question will depend on study type)
**Research aims, objectives, specific outcomes and hypotheses**		
Aims and objectives	Define the key aims of the study	What does the study ultimately aim to show?
		What are the primary and any secondary objectives?
Outcomes, interventions and predictors of interest	Identify the variables and quantities/qualities of interest that will be measured (these may be different for each hypothesis)	What is the primary outcome/response variable?
		Are there any secondary outcomes you also wish to measure and/or assess?
		What are the key interventions/groups/predictors you will be testing?
Research questions/Hypotheses	List the research question(s) that will be addressed and/or any hypotheses that you would like to test	The research question(s) should be defined in such a way that they
		- relate directly to the study objectives
		- relate to a specific outcome (or set of outcomes) and specific comparisons/predictors
		Each hypothesis should
		- be clearly testable
		- indicate what signifies a positive result for example, what is the minimum effect you would deem important?
**Study planning**		
Logistical considerations	Ethical approval	Will ethical approval be required for the study?
		- Will statistical support be required for the ethics application?
	Statistical support	What level of ongoing statistical support is available for this study?
	Data collection and management	How will the data be recorded and stored—will this require construction of a database?
		What steps will be taken to validate the data entered against what was collected?
		Who will be responsible for data entry and validation?
		Will any additional information (‘meta data’) be recorded to indicate data quality?
Materials and techniques	Laboratory equipment and methods	What specialist equipment and/or techniques will be used?
		Are there any aspects of these that may impact or limit the design of the study?
	Configuration and standardisation of materials and methods	Is there an accepted or validated way to measure the outcomes for this specific study or preliminary work be required to determine this?
		What are the possible sources of variation or systematic bias between samples/batches/observers/laboratories/centres?
		Are any aspects susceptible to systematic variation and/or bias? What steps will be taken to minimise measurement bias and variation with consideration to:
		- Technical factors—such as sample collection, processing, storage and analysis?
		- Biological factors—which may include the effects of comorbidities, diet, medications, stress, biological rhythms etc, on the measurement variable?
		Possible steps to consider in addressing these sources of variation might be the use of existing standards for sample processing or analysis (e.g., BRISQ, ISO, ASTM or CLSI), equipment calibration and maintenance, user training, randomisation of interventions.
	Software	What software (if any) will be used during data processing/collection/storage?
		What software will be used during data analysis—will specialist software be required?
		Does the software conform to any quality assurance standards, if applicable?
		Is the software up-to-date?
	What constraints/limits are there to the available resources?	What constraints are there? For example, due to cost and/or time
		- Are there any limits in terms of the available equipment (e.g., number of plates/chips) or materials (e.g., binding agents/gels)?
		- What would be the maximum number of samples that could be used/processed given the available resources and time?
**Study design**		
Design	Units of measurement	What are the sampling units in the study (e.g., blood samples from individuals)?
		Will the units be organised according to any structure (e.g., onto plates, chips, and/or into batches) or clustered/correlated in any way (e.g., samples from different centres), or within families, matched or paired samples/measurements?
		Will any repeated or replicate samples be taken? For example, any measurements over time; any biological replicates; any technical replicates.
		Are there any inclusion/exclusion criteria?
	Randomisation	Will any interventions or conditions be allocated at random to the units?
		- If so, how? (e.g., method of random allocation and process of generating random numbers)
		- If not, why not?
		Are there any other possible confounders (e.g., batches or plates) to which the units may need to be randomly allocated?
	Blinding (masking)	Will blinding be used? If not, why not?
		Who will be blinded and how?
		How will allocation be concealed and how will masking be maintained?
		Under what circumstances will the data be unblinded?
	Groups, treatments, and other predictors of interest	What are the primary groups or treatments of interest?
		What is your control or comparison group?
		Are there multiple independent variables to assess simultaneously (for example, treatment and time)? If so, will a factorial design be used (involving testing all levels of each variable with all levels of each other)?
		Are there any interactions of interest (which may, for example, lead to factorial designs)?
	Use of analytical controls	What analytical controls will be used? For example, qualitative (positive/negative) and/or quantitative quality controls (QCs); comparative/normalisation controls
		How will the controls be used/for what purpose?
	Other potential biases, confounders and sources of variability	Will you take any steps to minimise any background noise/variation?
		Will you measure and take into account any potential confounding variables? For example, the age and sex of any participants; batch/plate/chip effects; etc.
	Sample size considerations	Sample size will depend on the primary objective of the study, whether the aim is to test hypotheses, estimate a quantity with specified precision or assess feasibility
		Hypothesis testing:
		- Is there a single pre-specified primary hypothesis? Is a correction for multiple testing required?
		- What signifies a positive result (e.g., the minimum effect size, margin of agreement)?
		- What existing data are available to base the sample size calculation on? (e.g., SD of outcome)
		- What power and overall level of significance will be used? Will one or two tailed tests be used?
		Feasibility, pilot and proof of concept:
		- Understanding sources of variation (e.g., standard deviation of the outcome)
		▪ The sample size needs to be large enough to give an accurate estimates of any components of variation
		- Estimating with precision (e.g., proportion of samples that pass QC)
		▪ What is the acceptable precision (e.g., width of confidence interval) required?
		- Preliminary proof of effect (e.g., superiority of a new cell extraction technique)
		▪ What probability needs to be set to observe the correct ordering of your outcomes?
		▪ What level of significance would provide enough evidence to progress to fully powered study?
**Planned analysis**		
Data assessment and preparation	QC criteria	What pre-specified criteria will be used to assess data from quantitative analytical QCs?
		What pre-specified criteria will be used to assure the reproducibility of results?
		- Will any thresholds be set to screen or benchmark data quality (e.g., setting a maximum coefficient of variation that would be deemed acceptable)?
	Data verification	Have you allowed time for data validation and correction to be completed prior to analysis?
	Data normalisation/correction	Will the data be normalised or transformed in any way? If so, how?
	Outliers	What methods and criteria will be used to identify any outlying data?
Statistical methods	Describe the different analyses to be performed	Which models or tests will be used (e.g., t-tests; ANOVA; mixed effects models etc)?
		- Do these methods appropriately handle any repeated or correlated measurements?
		What assumptions do the statistical methods rely upon? How will these be assessed? Do the data require any transformation?
		Which comparisons will be made? For example, will all pairs of treatments be compared, or will each treatment just be compared to a control?
		What covariates will be adjusted for?
		If applicable, what model terms will be fitted, for example, which main effects and interactions, which fixed and/or random effects?
		Will sensitivity analyses be performed to assess the validity of the findings?
	Missing data	What might be the reasons for missing data?
		How will missing data be handled, for example, will missing data points be excluded or imputed?
	Multiple testing	Will a correction for multiple testing be required? If so, how many tests will be accounted for?
		Which adjustment for multiplicity will be used, for example, Tukey, Bonferroni, false-discovery rate
	Interim analysis	Will interim analyses be performed (before the full number of samples dictated by the sample size calculation has been collected)? If so, for what purpose (e.g., to update the required sample size)?
		Have any necessary adjustments to the sample size been made to account for the interim analysis?
	Replication and/or validation	Is there an intention to replicate the results (e.g., in an independent set of samples)?
		In there an intention to validate the results (e.g., using a different technique or method of analysis)?
**Reporting results**		
Guidelines/standards	Identify relevant reporting standards	What are the most appropriate reporting guidelines or standards that apply to the study design (e.g., BRISQ, MIFlowCyt and see www.equator-network.org). Identifying reporting standards at the planning stage helps to ensure that the information required to be reported is collected during the study and/or produced during the analysis of the data.

This framework is intended to support discussion within the research team as a whole, including the statistician.

## Research aims and objectives, specific outcomes and hypotheses

### Aims and objectives

The first stage of any study design should involve clarifying key details such as the aims and objectives, and the outcome(s) that will be measured. Early specification of the primary and any secondary objectives helps to ensure that the key goals can be appropriately addressed within a study by steering the necessary planning and resources towards tackling these issues. Often, multiple relevant and related objectives exist, but it may not be possible or desirable to adequately address them all within a single study. Resources, therefore, may need to be allocated according to the priority of each objective, and if any objectives cannot be adequately addressed it may be desirable to narrow down the focus of the study or to initiate further studies/collaborations to address the open issues. Note that decisions about which objectives should be prioritised over others may fundamentally impact on the best study design to use. The objectives need to be agreed upon at the outset to ensure the best and most efficient use of available resources.

Example(s): In the elastomer pump study in Box 1, the researchers ideally want to assess whether the new equipment performs as well as the existing equipment, and whether the performance of the equipment degrades over time. The amount of equipment available for use in the study is limited, however, so it may be sensible to prioritise one objective over the other unless both can be satisfactorily addressed with sufficient statistical power.

### Outcomes interventions and predictors of interest

The outcomes being measured should clearly relate to a study's objectives, and need to be chosen and prioritised accordingly. Primary outcomes are defined when undertaking hypothesis testing when the aim is to detect a specified effect. Secondary outcomes can also be tested, but the results from such tests will be interpreted as hypothesis generating rather than confirmatory. Any sample size calculation will be based on the primary outcome. If there are multiple primary outcomes a correction for multiple testing will be required, which will increase the required sample size for the study. Outcomes therefore need to be decided upon upfront, to ensure that an informed sample size calculation can be made.

Example(s): In the macrophage study in Box 2, the researchers want to assess the cumulative level of production for each of 10 cytokines over a 24 hr period. This study has 10 primary outcomes, and any sample size calculation would need to assume that (at least) 10 tests will be performed. The researchers also wish to compare levels between specific cytokines by measuring their ratios; these ratios may be viewed as secondary outcomes. If the estimated power for the study is too low (or, to paraphrase, the estimated sample size required is too large), the number of outcomes being assessed may have to be limited or reprioritised. A distinction should be drawn between the primary and secondary outcomes when reporting the findings from the study, with an acknowledgement that the assessment of the secondary outcomes may not be sufficiently powered.

### Research questions/hypotheses

Study hypotheses indicate how specific objectives will be addressed in a study, by spelling out the specific propositions and/or tests that will be assessed and how. The criteria used to address the objectives can have a major impact on all aspects of a study, from its design through to the interpretation of its results. Specifying the hypotheses upfront therefore ensures that these key details are decided upon at an early stage, and helps focus aspects of the study planning and design on tackling these questions.

Once at the reporting stage of a study, stating the hypotheses also plays an important role in preserving transparency about the full set of questions and/or tests addressed. All relevant hypotheses that were assessed should be reported regardless of whether the results obtained were positive or negative (or ‘null’). A distinction should also be made between the initially planned tests and any additional findings that were not part of the original test hypotheses. Exploratory and/or post-hoc analyses can play an important role in generating hypotheses for further study, but results based on these should generally be regarded with caution pending further validation. Alternatively a two-stage design could be used where exploratory findings can be investigated in new experiments within the same proposal. This approach is commonly encountered in ‘omics’ studies where a large number of variables are considered in the discovery stage and the ‘best’ of these carried forward for replication in new samples or validation in new experiments using a different method of measurement.

Note that some studies may not be designed to test a specific hypothesis; for example, pilot or feasibility studies aiming to establish and/or assess a novel assay. These studies, nevertheless, still have their own specific objectives, and these objectives need to be defined upfront (e.g., by clarifying what outcomes will be measured and defining any success/failure criteria).

Example(s): In the elastomer pump study in Box 1, the researchers aim to assess whether the new pump and catheter achieve acceptable flow rates over time. There are potentially numerous ways to define ‘acceptable’, such as a requirement that all flow rate measurements have to lie within 4 ml/hr ± 15% (i.e., 0.6 ml/hr), or allowing some measurements to lie outside these bounds so long as the mean flow rate lies within them. Alternatively, the researchers may prefer to test whether the new pump and/or catheter (or any combination involving the new pump or catheter) performs equivalently to the existing pump and/or catheter. In this latter scenario, an ‘equivalence test’ might be performed. Equivalence tests usually assess an alternative hypothesis that a new and an existing intervention are equivalent (versus a null that they are not) by measuring whether the difference in means between the two interventions (and its confidence interval) lies within pre-specified particular limits. In this study, the hypotheses may therefore be laid out as follows:

H_0A_: The 95% confidence interval for the difference in mean flow rates between new and existing pumps does not lie within 0 ml/hr ± 0.6 ml/hr.

H_1A_: The 95% confidence interval for the difference in mean flow rates between new and existing pumps lies within 0 ml/hr ± 0.6 ml/hr.

H_0B_: The 95% confidence interval for the difference in mean flow rates between new and existing catheters does not lie within 0 ml/hr ± 0.6 ml/hr.

H_1B_: The 95% confidence interval for the difference in mean flow rates between new and existing catheters lies within 0 ml/hr ± 0.6 ml/hr.

H_0C_: The 95% confidence interval for the difference in mean flow rates between any combination of new pump and/or catheter and the existing pump and catheter does not lie within 0 ml/hr ± 0.6 ml/hr.

H_1C_: The 95% confidence interval for the difference in mean flow rates between any combination of new pump and/or catheter and the existing pump and catheter lies within 0 ml/hr ± 0.6 ml/hr.

These hypotheses confirm the key (primary) questions of interest that will be tackled within the study, illustrate how the interventions will be assessed, and define the criteria by which to discriminate between positive and null results.

## Study planning

### Logistical considerations

This section of the framework addresses aspects of the study which might impact on the extent of statistical support required. In some cases, scientists may have limited access to a statistician, and whilst we would argue that statisticians should play an integral role in the research team, we accept there may be some instances in which the opportunities for them to provide advice and input are rare. Therefore it is useful to consider early on whether statistical support might be required during the planning and conduct of the study. If there is limited statistical support then this may limit the complexity of the analytical approach that can be recommended.

Giving early thought to the means by which data will be collected and managed during the study can be vital to reproducibility, whilst also impacting on the resources required. Constructing a well-designed, fully validated database should ensure good quality data are collected and may reduce delays in detecting errors. Collecting additional data regarding data quality (sometimes referred to as ‘meta-data’) can be helpful to the statistician at the analysis stage. For instance, it is a good idea to indicate the reason why a data value is missing.

Example: In the study in Box 3, it would be a good idea to collect meta-data regarding the batch numbers and date(s) on which the samples were processed.

### Materials and techniques

The design of a study clearly depends on the materials and equipment available for use. All studies have resource constraints and, as described in section A, these need to be discussed in order to ensure that the key hypotheses can be appropriately addressed. Other restrictions concerning the materials and equipment can also impact on study design.

#### Laboratory equipment and methods

Financial constraints are the most commonly encountered limiting factor, which in turn may lead to limited access to facilities. However, particular equipment may also be limited in terms of the number of units that can be processed within the available timeframe and/or in a particular batch. If the equipment or resources available for use are heavily constrained and not sufficient to provide an adequate sample size for the primary research question identified in section A it may be preferable to revisit and redefine the study's objectives, hypotheses and/or outcomes to be measured in some other way, rather than carrying out an underpowered study.

Example: Box 1 presents a study where the number of units of equipment available to test is strictly limited. The experimenters could consider redefining how they assess an ‘acceptable’ flow rate (e.g., specifying a minimum number of measurements that must fall within set boundaries, rather than testing for equivalence or statistically significant differences). Alternatively, the researchers may decide to go ahead with the study as originally planned, with the acceptance that it will be unlikely to deliver a conclusive answer to the primary research questions. In this scenario, the study could serve to generate pilot-data to assist the planning of a future follow-up study, and/or to contribute a wider meta-analysis of other, sufficiently similar studies.

#### Configuration and standardisation of materials and methods

Processing samples in different batches or across different pieces of equipment frequently introduces technical variability into a study, yet is often unavoidable. These potential sources of variability need to be anticipated and even studied in advance, and steps taken at the design stage to avoid confounding technical variation with any particular groups or comparisons of interest (see later sections on ‘Other potential biases, confounders and sources of variability’ and ‘Randomisation’).

Equipment and/or experimental methods and procedures may need prior validation before use within a study. Appropriate configuration of methods and equipment can help to minimise unwanted variation between different experiments and units and, hence, ensure that measurements generated in a study are sufficiently accurate and reproducible. Other factors such as appropriate maintenance of equipment or training of staff to use specialised equipment may also impact.

There are a number of organisations that provide information to help researchers identify appropriate means of performing quantitative and qualitative quality assurance. In particular the World Health Organisation laboratory quality management system training toolkit is a comprehensive and freely available online resource (http://www.who.int/ihr/training/laboratory_quality/doc/en/). Guidelines and standards are also available from the Clinical and Laboratory Standards Institute (http://clsi.org/standards/) and the US Food and Drug Administration (http://www.fda.gov/downloads/Drugs/Guidances/ucm070107.pdf). These guidelines are routinely used in accredited industry and medical laboratories and provide valuable information about many ‘gold standard’ laboratory practices.

Example: In the macrophage example in Box 2, the bead arrays require prior validation; to do this, external information about typical standards for the equipment (such as acceptable coefficients of variation) may need to be sought and/or determined. As the configuration of equipment often affects the variability of measurements recorded within a study, any validation steps may also impact on sample size and power calculations.

## Study design

### 

#### Units of measurement

Experimental units are the entities that receive a given ‘treatment’; it should be possible for two different experimental units to receive two different treatments of study conditions. Sampling units are the entities upon which measurements will be made. The experimental units can usually be considered to be independent of one another, so increasing the number of experimental units measured in a study usually increases the amount of independent information sampled. In contrast, any repeat or replicated measurements taken on the units do not contribute additional *independent* information, but can nevertheless help to gauge measurement uncertainty and/or stabilise estimates of inherently variable measurements. Repeated measurements may also be used to answer additional questions of interest. An important consideration concerning the experimental units is the definition of any inclusion or exclusion criteria.

Example(s): In Box 1, each combination of a specific pump and a specific catheter on a single equipment bench makes up an experimental unit (see Box 1 Figure 1). There are four benches of measuring equipment on each of which four different combinations of new/old pump and new/old are tested, to produce 16 experimental units. Each unit is tested three times to give three replicate experiments. During each replicate experiment, measurements will be taken on the units at 2 hr intervals over 48 hr periods; each individual measurement made during each experiment can be considered a sampling unit. The sampling units will help to provide precise estimates of the mean flow rate in a given experiment, and may also contribute information about whether the equipment degrades in performance over time. However, since they are all collected from the same experimental unit, they cannot be considered independent of each other; failure to correct for this in the analysis would artificially inflate the power of the test and potentially give misleading results (we expand on this issue below under ‘Statistical methods’: Describing the different analyses to be performed).

In the study in Box 3, the sampling units refer to samples taken from individual volunteer donors. A single sample is taken from each donor, so in this case the sampling units are independent of each other and the sample size for the analysis is the total number of sampling units. The aim is to compare gene expression between hypertensive and normotensive individuals; therefore, both hypertensive and normotensive must be defined along with any other restrictions on co-morbidity or age and gender.

#### Randomisation

Randomisation plays a crucial role in protecting studies from known and unknown sources of variation, bias and confounding. Moreover, implementation of an appropriate randomisation strategy can also begin to produce evidence of causality in experiments. Randomisation is already widely used in clinical trials during the allocation of treatments to units, but it serves the same fundamental purposes in laboratory settings involving the direct manipulation of any experimental treatments or conditions. Although implementing a randomisation scheme can be cumbersome to employ and may involve added complexity within a study, the potential benefits it provides offer researchers protection against future claims of unconscious bias and should directly lead to enhanced reproducibility. A randomisation plan should therefore be devised wherever possible.

While randomisation is a simple concept in principle, in practice it may need to be employed as a joint component of the design implementation. In the simplest case where there are no groupings or balancing factors to consider, a simple randomisation procedure can be employed. If the experiment needs to be conducted in batches then randomisation should be employed within each batch with a balanced number randomly selected to each treatment group in each batch. The same consideration needs to take place in a study using case control samples with a random selection of cases and controls to each batch. More complicated designs with two factors (e.g., treatment group and time) such as the Latin square, use random permutations of rows and columns to maintain the balance.

Note that randomisation can also play an important role even in studies that do not involve any direct manipulation of experimental conditions or interventions. For example, in observational studies the effects of potentially confounding factors such as batch effects can be alleviated via careful use of randomisation.

Example(s): The study in Box 3 aims to analyse kidney tissue samples from hypertensive and normotensive patients using RNA sequencing. RNA-sequencing may be susceptible to batch effects, however, so care should be taken to randomise both case and control samples to each batch to avoid confounding any potential differences in gene expression between cases and controls with any differences between batches.

The way that macrophage differentiation is shown in Box 2 Figure 1 would suggest that conditions may vary if donors are processed in a series rather than in parallel. However, this experimental design does control for between batch variation as each donor's differentiated macrophages are infected and treated concurrently.

In the study in Box 1, there are multiple ‘treatments’ (i.e., combinations of new/existing pump with new/existing catheter) to test on each of the four equipment benches. This is an example of a study where it may be desirable to manually control the order in which units receiving each treatment are tested rather than using a fully randomised design. For instance, Box 1 Figure 1 shows one potential, manually allocated design, in which every combination of pump and catheter is tested across the four benches at any one time, and where the order of running the combinations is different on each bench. This design avoids biasing measurements on any particular combination due to any potential time-dependent effects/drift (i.e., as all combinations are always tested at the same time); in addition, it allows each combination to be tested with both the unused and used version of each pump, and both the unused and used version of each catheter. Note that although this arrangement is not strictly random, a random process may be used to select which components are used together at the starting point. Alternative arrangements, such as completely randomising the combinations over the benches, or manually arranging the combinations without regard to potential confounders (e.g., at the convenience of the experimenters), would be unlikely to balance the combinations over all potentially confounding factors in this relatively small scale study, and may be inferior to a carefully planned, manually allocated design.

#### Blinding

Blinding (or ‘masking’) aims to guard against potential bias within a study by concealing information about the allocation of treatments or interventions from the individuals involved—such as patients, experimenters and/or analysts. Awareness of the true allocation of treatments may consciously or unconsciously influence the behaviour of those involved, thereby biasing evidence in favour or one treatment over another. Blinding is especially important if qualitative judgement makes up any part of the measurement process.

Example(s): In example study 1, blinding may be implemented by concealing the pump and catheter types, if possible, from the experimenter involved in setting up the equipment. Any study analysts may also be blinded, for example, by using codes to reflect intervention types in the resulting datasets. Note that it may not be possible to fully blind everyone involved in this study, particularly if the two types of pumps and/or catheters in Box 1 have obviously different appearances. In this scenario, one potential way of maintaining the blinding would be to conceal which of the pumps and catheters are the new and existing models (and, therefore, which are the experimental treatments and which are the controls). Nevertheless, even if the experimenters cannot be blinded in this study, plans should be put into place to blind any analysts involved.

In the study in Box 2, the experimenter should ideally be blinded to the infection status of the cells and to the treatment type.

#### Groups, treatments and other predictors of interest

Most studies involve making at least one form of comparison between groups or interventions of interest. Comparator groups—usually called ‘control’ groups—may be positive or negative in nature (i.e., active or inactive respectively), depending on the aims of the study. For instance, a negative control group may be included to assess whether an experimental treatment has a greater effect than a placebo, while a positive control group might be used to assess whether the experimental treatment is superior to an existing treatment. These controls, data from which contribute to statistical assessment of the research question, are distinct from analytical controls used during data collection to check that laboratory processes are running as expected (see section on ‘Use of analytical controls’, below).

Often, it may be of interest to compare experimental groups under different conditions or alongside one or more additional factor of interest. Where studies contain more than one factor of interest (including the main experimental groups), they may be considered to have a ‘factorial’ design if all combinations of the levels of each factor are tested. Factorial studies provide an efficient means of examining the effects of multiple factors within a study, because each experimental unit contributes information towards all factors of interest. In addition, they also enable the potential effects of interactions to be investigated, which allow the effects of one variable to differ depending on the value of another.

Example: The Box 2 example may also be considered a factorial experiment, because it assesses the effects of both bacterial infection and drug treatment on macrophage activity simultaneously. Here, the factorial nature of the study allows the researchers to assess whether the effect of the drug differs depending on whether the cells are infected with bacteria (i.e., whether there is an interaction between drug treatment and bacterial infection). In this study, each factor of interest (‘bacterial infection’ and ‘drug treatment’) is to be validated against a negative control (‘mock infected cells’ and ‘no treatment’ respectively). The controls here serve to enable claims to be made about any potentially causal effects of the factors of interest. For instance, if the drug treatment was compared to a pre-treatment or baseline measure instead of a control, no information about what could or would have happened in absence of treatment would be available (for example, perhaps macrophage activity could have changed naturally between the two time-points).

#### Use of analytical controls

Analytical controls tend to be used to validate practices within an experimental assay, helping to ensure that measurements are accurate and may be interpreted correctly. Analytical controls may be required for each variable or condition in the experiment, for quality control (QC) purposes and/or to gauge and adjust for background variation that may systematically influence certain sets of measurements (see Table 2 and the ‘QC’ section for further details).

**Table 2.** Commonly encountered examples of analytical controls **DOI:** http://dx.doi.org/10.7554/eLife.05519.011

Control type	Purpose
QCs	Qualitative QCs typically indicate whether specific aspects of the experimental and/or analytical procedure work in the intended ways, and are often included in the same analytical run used to collect study data. For example, a negative control may be a sample or unit that is known to be negative for the outcome and, hence, should assign a negative measurement in the assay. In contrast, a positive control would be expected to assign a positive result.
	Quantitative QCs are used to monitor the performance of a quantitative measurement system and ensure that it is performing within acceptable limits. Typically quantitative QC samples are run at two or more concentrations across the range of the assay and interpreted using graphical and statistical techniques, such as Levy-Jennings plots and Westgard rules. QC materials are generally not used for calibration in the same process in which they are used as controls.
	In instances where any QC checks fail, certain aspects of the experimental procedure may have to be altered in order to remedy the problem or one or more units associated with the violation may have to be reprocessed until satisfactory checks are achieved.
Comparative/normalisation controls	These can be alternative physical or biochemical parameters measured alongside the analyte of interest usually within the same sample, for the purposes of normalisation and/or correction. For example, in RT-PCR housekeeping genes are usually amplified as well as targets of interest, with the final output expressed as a ratio between the target and the housekeeping gene.

Example: In the elastomer pump study in Box 1, temperature measurements made during data collection can be used as a form of normalisation control to obtain temperature-adjusted estimates of flow rate.

#### Other potential biases, confounders and sources of variability

Potential sources of bias and variability need to be anticipated upfront—at the design stage of a study—in order to avoid or account for their effects. Systematic sources of variation can often be tackled via careful study design; for example, by balancing and/or randomising treatment arms over potentially confounding variables (such as plates or batches, or having multiple observers/experimenters involved in data collection). Similarly, potential biases may be avoided by ensuring experimental runs are conducted under homogeneous conditions wherever possible (such as under a fixed temperature), and that measurements are consistently made (e.g., by using properly calibrated equipment). If any unwanted sources of variation cannot be controlled, it may be possible to adjust for their effects during analysis if the key variables are measured during the study (Stegle et al., 2012). Note that, as suggested above, an additional source of variation may occur where multiple researchers are involved in conducting an experiment or in any aspect of the measurement. This is often seen as a negative aspect of an experiment where the goal is to reduce error as far as possible. However, one positive aspect of this is that results can give an indication of how robust the experiment is in a wider context. Ultimately, some level of variation should be anticipated to occur amongst operators or sites and this needs to be reported and accounted for (Barnhart et al., 2007; Maecker et al., 2011).

Example(s): In the Box 1 example, temperature cannot be controlled between experiments or time points, but plans have been made to measure it concurrently with the flow rates. As such, any confounding effects of temperature can be controlled at the analysis stage by including temperature as a covariate. As this study has a hierarchical design (i.e., where measurements will be taken on units across multiple experiments and over sequential time-points within an experiment), there will also be multiple sources of variation that need to be accounted for during analysis (such as ‘between time-points within an experiment’ and ‘between experiments on the same unit’).

#### Sample size considerations

Sample size calculations aim to establish the minimum sample size that a study requires in order to be in a strong position to answer the primary research question. The primary research question may take the form of a statistical hypothesis test, an estimate with specified precision, or to obtain evidence for proof of concept (POC). With a statistical hypothesis test the aim is to control for two forms of error; type 1 in which the null hypothesis is rejected when it is true (false positive), and the type 2 error in which the null hypothesis is not rejected when the alternative is true (false negative). The most common error levels to adhere to are 5% for a type 1 error and 10% or 20% for a type 2. When the type 2 error is 20% we have an 80% chance (or power) of rejecting the null when the stated alternative is true. In the precision context, the aim is to estimate a population parameter of interest such as the standard deviation of an outcome, or an event or prevalence rate. In this form of study, the aim is to control the expected standard error of the estimate derived from the sample. POC studies are typically conducted to obtain some preliminary evidence that a treatment/intervention works. One approach is to calculate the sample size that will give a sufficiently high probability (90–95%) to observe the correct ordering of the primary outcome of the treatment/intervention and control group. If the estimate for the primary outcome is favourable for the treatment/intervention group then this would support a decision to continue with a larger hypothesis testing study (Piantadosi, 2005).

Sample size calculations rely on various conditions and assumptions. We need to state which assumptions we have made and justify why it is fair to make them. In a hypothesis testing framework, once we have identified the form of the primary outcome (e.g., binary, continuous, or time to event) and how we propose to compare the groups (e.g., a relative risk; difference between group means; hazard ratio, etc) we can discuss what the minimum important effect size might be. Deciding upon the magnitude of effect size to use in a sample size calculation can be difficult. The most common strategy involves attempting to define the minimum meaningful difference. This approach does not require prior knowledge as the investigator should choose the smallest effect size they would be willing to miss (if there was a true difference). This can be an inherently subjective task, and an effective strategy may involve estimating the required sample sizes over a range of possible effect sizes.

For common and well-studied clinical outcomes such as blood pressure or body mass index, the variability of the outcome in the population being studied (as well using the planned means of measurement) are usually well established. If researchers do not have data on the outcome of interest then it may sometimes be possible to obtain estimates of variability from similar published studies. Using the literature to inform a sample size calculation can be more convenient than performing a pilot study and, if multiple suitable estimates are available, this will provide a range for the expected level of variability. Nevertheless, external estimates of the variability may not necessarily be directly comparable to the potential level of variability in a new and independent study—especially where there are differences in procedure and/or methods of measurement.

Studies that involve any repeated and/or replicated measurements on each unit are influenced by multiple sources of variation. For instance, measurements taken across experimental units over time are influenced by ‘between time’ and ‘between unit’ components of variation. Any sample size calculation for a study involving repeated or replicate measurements therefore requires estimates of each variance component in order to accurately predict the required sample size. In complex study designs involving multiple sources of variation, it is unlikely that estimates of all applicable variance components will be available from the literature. A pilot study of interim analysis of the data may therefore be required in order to provide a meaningful estimate of the required sample size (see ‘Interim analysis’ section). Sample size calculations for these studies, by definition, may also be more complex, often requiring a computationally intensive method such as estimation by Monte Carlo simulation.

Example(s): The study in Box 1 plans to take repeated measurements on each experimental unit over time, and to test each combination of components in triplicate. Each additional measurement of the flow rate adds information to the study and will, up to a certain point, help to increase the statistical power of the study. An estimate of each source of variance would be required to accurately estimate the power (or required sample size) for this study, which may not be readily available in previous publications. As such, a pilot phase might be built into this study in order to inform a sample size calculation (see ‘Interim analysis’ section later). In addition to estimates of the applicable ‘variance components’, any sample size calculation would also require a definition of the desired type I and type II error rates. Furthermore, the ‘minimum meaningful difference’ would also need to be defined. As this study may be conducted as an equivalence test, the minimum difference might be taken as the ‘equivalence limits’ in which the 95% confidence interval for the difference in flow rates must lie (i.e., previously defined as ±0.6 ml/hr).

## Planned analysis

### Data assessment and preparation

#### QC criteria

QC procedures aim to assess the validity of any data collected in a study, and to detect any errors that may have occurred, thereby helping to avoid the potential effects of any biases or unwanted variation that may arise. Often, QC procedures involve analysing control samples included in the design of the study (see ‘Use of analytical controls’ section). Plans for handling data from any analytical controls therefore need to be defined upfront so that any experiments or samples that fail QC can be repeated or reanalysed if required.

Criteria may be set to verify that any measurements taken within a study are sufficiently accurate. Westgard's rules (Westgard et al., 1981) are an example of multi-rule criteria used to determine whether an analytical run is out of control.

Another reason to set criteria is, to check whether data from calibrators, analytical controls or study samples are reproducible. Thresholds for any such criteria must be set a priori using benchmarks from any preliminary or published work, on the premise that if an experiment or set of measurements does not satisfy these criteria, components of the study may have to be repeated or certain data points excluded.

Example(s): In the macrophage study data are collected at multiple time points. Results may fail QC at any one of the measurement time points and in any assay batch. The cause of this failure may be due to a whole plate being contaminated before the assay, or due to a technical fault of the measurement system. The impact of a failed plate when longitudinal measurements are made may be larger as this prevents further measurements being made and calls into question prior measurements before the contamination was detected. So a full or partial repeat of the whole experiment may be necessary. The failure of a single assay batch may be more recoverable depending on the proportion of missing data in measurements needed at that time point. In the described design there are two replicates so a sensitivity analysis could be employed in which extreme values (e.g., single measurements more than 3 SD away from the batch specific mean) are coded as missing.

#### Data verification

Where necessary data in the database for analysis should be checked against its source to identify data entry errors prior to analysis. This important step can take time and should be incorporated into the analysis plan.

#### Data normalisation/correction

Other aspects of data preparation may involve attempting to correct for potential problems such as known (or unknown) biases or confounding effects. Normalisation methods are often used to align data to an expected distribution, with the aim of ensuring that the groups being tested are comparable. This can involve taking into account information on the structure of the study design such as batch or centre numbers or by using data from appropriate analytical controls. The planned normalisation or correction procedure may have implications for the subsequent analysis of the data and should be specified in advance.

Example(s): The study in Box 3 involves several stages of sample and/or data processing, each of which may require implementation of specific QC procedures. For instance, RNA quality and the possible impact of DNA contamination need to be assessed, with criteria potentially set to exclude bad samples (e.g., using the RNA integrity number score). The processes involved in quantifying the transcriptome (e.g., using the Tuxedo suite of software) may also be subject to data quality issues and need to be assessed accordingly. As RNA-sequencing can be inherently susceptible to batch effects and/or other unwanted sources of variation, data correction techniques such as PEER (Stegle et al., 2012) may also be employed to normalise data profiles across samples.

#### Outliers

Having performed appropriate checks that the data are accurate and reproducible, it is good practice to use a combination of descriptive and graphical methods to assess the distributions of your study variables to check for outliers. It is not good practice to routinely discard such outliers from analysis; however, having performed the primary analysis on the full dataset, one can perform sensitivity analyses that exclude outliers, to show how they might be influencing the conclusions. Where possible the criteria for identifying potential outliers should be specified in advance of obtaining the results.

### Statistical methods

Early consideration of the statistical methods helps to ensure that a study's objectives will be reliably addressed. It allows study design to be optimised by enabling an appropriate sample size calculation to be made, and ensures that the resulting data will be suitable for the most appropriate statistical analysis. Specifying firm details about the anticipated statistical methods upfront, including the analytical strategy for any secondary research questions or potential subgroup analyses, can also help to avoid biases at the analysis and reporting stages. In particular, it helps guard against the selective reporting (or ‘cherry picking’) of favourable results, and provides full transparency about the initial analysis plan. A further advantage of clarifying details about the planned statistical analyses upfront is that, where applications for funding will be submitted, it may provide an opportunity to cost in time for any necessary statistical support that will be required, such as for regular integrated discussions with a statistician or for the dedicated statistical analysis. This section of the checklist details the key analytical considerations that should be decided upon upfront during study planning.

#### Describe the different analyses to be performed

The methods that will be used can fundamentally impact on the types of inferences that can be drawn from a study. As such, these should be decided upon upfront, along with related details such as any model terms or covariates that will be considered and the specific tests or comparisons that will be performed. If data require a transformation prior to the analyses then all such transformations need to be documented and clearly justified. These aspects of the statistical methodology all have implications for the sample size calculation, and can influence the scope and the validity of the findings. As different statistical methodologies rely on different assumptions, plans to assess the validity of these assumptions should also be made. If any of the assumptions do not hold then the results of the analysis may be misleading. For such situations it may be that a simple data transformation will suffice, if not alternative methods may be required for which additional statistical support may need to be sought. Sensitivity analyses can provide a means of assessing the dependency of research findings upon the assumptions, and can help to strengthen any conclusions being made.

Example(s): The study in Box 1 measures flow rates over time on each pump-catheter combination, and plans to replicate each experiment three times on a particular experimental unit. As such, the measurements collected in this study are not independent; flow rates recorded close together in time may be more similar than those recorded at different times, whereas the measurements gained in a particular experiment or unit may be more similar than those measured across experiments or units. Many conventional statistical methods assume that all observations are independent and, hence, may produce misleading results if applied in this study and pseudo or false replication occurs when there is such a mismatch between the experimental design and the statistical analysis method (Hurlbert, 1984). An appropriate method for handling repeated measurements would instead be required, such as a mixed-effects model. Mixed-effects models handle non-independent measurements (sometimes referred to as ‘pseudo-replicates’) by including ‘random effect’ terms. Any parameters or factors of interest that need to be tested—such as the pump and catheter effects—would be included as ‘fixed effects’. After fitting such a model, planned comparisons can be made to assess the key hypotheses; for example, to quantify: (1) the difference between new and existing pumps; (2) the difference between new and existing catheters; and (3) the difference between each combination involving a new pump and/or catheter and the combination of existing pump and existing catheter.

#### Missing data

Planning to handle any missing data that may arise upfront can help to avoid potential problems and bias at the analysis stage. Missing data may arise for any number of reasons, but any obvious problems that could occur should be anticipated in advance and plans made to deal with their possible effects. Depending on the study design, it may be possible to guard against missing or inaccurate data by monitoring data quality as it accrues; pilot studies are a good way of identifying potential issues before the full study begins.

Example: In the elastomer pump example, measurements were to be made automatically over a period of 48 hr. If for any reason the equipment were to fail during this period, longitudinal data would be missing from the point of failure onwards. In this example, use of a mixed-effects model would allow for the inclusion of incomplete longitudinal datasets; in contrast, if an alternative method such as repeated-measures ANOVA were used, sets with missing data would have to be excluded, reducing power, or the missing values would need to be imputed, possibly introducing bias depending on the methods used.

#### Multiple testing

Running multiple tests within a study usually requires some form of correction for the number of tests being made (often referred to as accounting for ‘multiplicity’). This guards against the increased chance of obtaining positive results just by chance as you increase the number of tests or observations being made on the same data. A type 1 error rate of 5%, that is, testing at p < 0.05, suggests that 1 in every 20 tests will be significant simply by chance. The two most commonly used forms of adjustment involve controlling either the ‘family-wise error rate’ or the ‘false-discovery rate’ (FDR). The family-wise error rate assumes a given probability of obtaining one or more false-positive results within a set (or ‘family’) of tests. Often, a 5% family-wise error rate is used—meaning that, on average, only 5 out of 100 repetitions of the complete set of tests would contain at least one false-positive result. In contrast, the FDR assumes—usually less stringently—that a given proportion of a particular set of positive results are false-positive. Deciding upon the means of adjusting for multiplicity—including defining the number of tests to adjust for and/or what constitutes a single family of tests, can be a contentious issue.

Example(s): In the study in Box 2, 10 cytokines will be tested, and multiple comparisons of treatments will be made for each cytokine. A suitable adjustment for multiplicity would therefore account for the number of comparisons of treatments made for each cytokine, and the number of cytokines tested.

In the Box 3 example, a large number of transcripts will be tested for association with hypertensive status, creating a multiple testing issue. Many of the transcripts are expected to be highly correlated with one another, however, while most adjustments for multiplicity assume that all tests being corrected for are independent. In this scenario, adjusting for the full number of transcripts tested would be conservative, and could—arguably—unfairly reduce the statistical power of the study. As such, it may be reasonable to use a less conservative adjustment in this study, or to seek a more sophisticated approach that can better account for the number of independent tests being made.

#### Interim analysis

Properly planned interim analyses can strengthen the quality of the data and/or reduce costs, because they potentially allow for the sample size calculation to be updated with more accurate information, or for data collection to be stopped early. However, they must be planned in advance; ad hoc analysis of data before the final sample size is reached risks falsely rejecting the null hypothesis, due to multiple testing or to obtaining a biased estimate of the effect size in too small a sample.

Having discussed the study design with reference to the framework, there may be elements that cannot be addressed immediately with confidence. For example, data underpinning the sample size calculation may be of uncertain quality/applicability, or suggested adjustments to the methods may need to be trialled for feasibility. An interim analysis after a certain proportion of the data had been collected would allow adjustments to the sample size to be made, or potentially would allow data collection to stop altogether. Under some circumstances interim analysis would require breaking of a blind, or inflation of the final sample size required. For this reason interim analyses should be planned fully in advance, with consideration given to the practical implications of performing the analysis, and rules should be defined which determine the circumstances under which data collection should continue.

Example(s): The study in Box 1 has not been subject to a formal sample size calculation due to a lack of available data on the magnitude of the various components of variation planned into the design (e.g., the variation in flow rates between time-points in a particular experiment on a particular unit, the variation between experiments, and the variation between units). As such, it would be desirable to plan for an interim analysis of the data during the study in order to estimate the sample size required to run any equivalence tests with sufficient power. If the interim analysis solely involved estimating variance components, it would not be necessary to break the blinding of the interventions or add to any multiple testing burden. However, if the experimenters wished to assess for equivalence at an interim stage of the study, the planned sample size would need to be increased further in order to properly allow for this. Note that, contrary to these plans, this entire study may instead be considered to be a pilot for a larger future study. In this scenario, it may not be worth conducting any interim analyses; the resources planned for the current study may already be fixed, with no scope for increasing the sample size if required.

#### Replication and/or validation

Validation and/or replication of the results provides valuable support to research findings. Validation usually involves using a different method and/or technique to confirm data that has been obtained—it thereby helps to guard against any biases or confounding associated with measurement and/or processing. In contrast, replication usually refers to reproducing results in an independent dataset (such as an additional set of samples that were not included in the original analysis). Replication can help guard against confounding associated with the experimental/sampling units, and also protects against statistical issues such as ‘overfitting’ and ‘The Winner's Curse’.

Example(s): The study in Box 3 may be considered a ‘hypothesis generating’ study whereby it aims to identify genes and biological pathways that may be associated with hypertension. Findings from hypothesis generating studies, by definition, require subsequent confirmatory work in order to reaffirm any findings. Confirmation of findings may be achieved by replicating any positive results in an independent study or an independent set of patients. Validation of the data may also be desirable, particularly if any QC checks highlight any potential problems with the data such as batch effects. This may be achieved, for example, by reanalysing any interesting genetic variants using another technology such as a genotyping array.

## Reporting results

Once a well-designed laboratory study has been completed, it will need to be reported to a high standard to enable future reproduction of the results. There are a wide range of publications available which give detailed instructions on how best to report the results of different types of studies. Many journals now require authors to refer to specific guidelines for certain research designs.

It is not our intention to give an exhaustive list here, as new or updated guidelines are released with regularity. However, authors should search for relevant guidelines when preparing for publication; the Enhancing QUAlity and Transparency Of health Research (EQUATOR) network website is an excellent place to start, featuring a searchable library which aims to include all reporting guidelines published since 1996 (www.equator-network.org).

Example(s): If the elastomer pump study in Box 1 was treated as an equivalence study then many of the recommendations in the CONSORT extension for equivalence clinical trials would be relevant.

## Summary

The RIPOSTE framework aims to reduce irreproducibility in laboratory based research by encouraging early discussion of study design and analysis within a multidisciplinary team including statisticians. We seek to steer discussions within research teams towards addressing key aspects of experimental design and analysis at the earliest stages of a study, and believe that this increased focus on planning will lead to more rigorous research and ultimately reduced wastage in preclinical research.

Lack of reproducibility is not the sole reason for wastage within laboratory studies. In January 2014 the *Lancet* printed a special issue focussing on how to increase value and reduce waste in medical research (Macleod et al., 2014). It has been claimed that much of the waste is due to incomplete and unusable results (Chalmers and Glasziou, 2009). The problem of poor research practice and documentation is widespread and entrenched in the scientific culture (Collins and Tabak, 2014). Currently scientific rewards are disproportionately high for being the first to publish, and this pressure has played a major part in generating the problems with reproducibility that are now being highlighted (Begley, 2013; Ioannidis, 2014).

A number of recent initiatives have drawn further attention to these critical issues and proposed strategies to address and change the scientific culture. The common themes emerging from these initiatives are to improve training on experimental design and analysis, to involve experienced statisticians at all stages of design and analysis, to raise awareness at grant review stage of aspects of design such as randomisation and blinding, and to reward good quality, well designed research. Ioannidis et al. (2014) make three broad recommendations to improve study design, conduct and analysis. The first of these is to make study protocols publicly available including the raw data and analytical algorithms. The second promotes raising the profile of defensible research proposals within well-trained research teams. The third is to reward reproducible practices through funding and academic recognition. In the same special issue of the *Lancet*, Glasziou et al. (2014) recommend that funders should support and encourage their research institutions to share research protocols and study materials and ultimately to promote high quality complete reporting. At publication the emphasis must move towards reporting results in which they have confidence (these will often be negative) in detail, rather than selectively reporting the details of the positive results which, if spurious, will serve to misguide the research community. Several recent incentives to promote direct replication research are beginning to make an impact with the publication of registered reports (Nosek and Lakens, 2014). In this framework journals agree to accept a future publication based on acceptance of pre-registered proposals and prior to any data generation.

In addition to the above initiatives, there has also been a recent push for greater publication of raw data. The PLoS journals, for example, implemented a new data policy earlier this year stipulating that authors must, wherever legally and ethically possible, share all data, metadata and methods that underlie any research findings offered for publication (Bloom et al., 2014). Data must be deposited in a public repository, uploaded online in supporting files to accompany a manuscript, or made available upon request; any failure to ensure that sufficient provisions to share have been made can be grounds for rejection. In the US, the NIH also intends to promote greater access to raw data, requesting that funding applications include a Data Discovery Index to enable any unpublished data to be more easily located, accessed and referenced by other researchers in any future work (Collins and Tabak, 2014). This NIH initiative has been supported by recent calls to prospectively register laboratory studies (Hooft and Bossuyt, 2011; Altman, 2014). Registering studies upfront would necessitate that any deviations from protocols are both documented and justified, and would ensure that protocols are well thought out at an early stage (i.e., prior to registration). It would likely also significantly improve the transparency of research, as was seen following the implementation of a similar initiative in 2005, which sought to introduce a requirement to prospectively register certain types of trials (Hooft and Bossuyt, 2011). Registering studies and reducing bias against publication of negative results will also help to ensure that replication studies with negative findings receive the appropriate attention amongst the scientific community.

These initiatives suggest the need for a major culture change within preclinical research; tackling these issues will require effort on multiple levels. The shift to making statisticians an integral part of the research team rather than to be consulted in isolation will be challenging. Statisticians' knowledge and experience of experimental data and the laboratory environment are highly variable. Scientists may be reluctant to work with statisticians in this way due to variable experiences in the past. The RIPOSTE framework has been designed to support this shift and help scientists and statisticians alike form a deeper understanding of the issues surrounding the reproducibility of laboratory research. This should ensure that the considerations relevant to a particular study can be addressed efficiently with greater confidence on both sides. To allow for a greater involvement of statisticians in the study design process, additional funds will be needed and this will require commitment from funding bodies. We recommend that statisticians be considered an integral part of the research team wherever possible, and that they should be involved at the planning stages of studies. We encourage use of our framework for all laboratory research studies and not just those seeking funding. In conjunction with other initiatives (Collins and Tabak, 2014) the RIPOSTE framework can be a useful tool in combating irreproducibility of preclinical study results, offering a powerful riposte to the criticisms regarding wastage in laboratory research.

## References

[bib2] Altman DG (2014). The time has come to register diagnostic and prognostic research. Clinical Chemistry.

[bib1] Altman DG, McShane LM, Sauerbrei W, Taube SE (2012). Reporting recommendations for tumor marker prognostic studies (REMARK): explanation and elaboration. BMC Medicine.

[bib3] Baggerly KA, Coombes KR (2009). Deriving chemosensitivity from cell lines: forensic bioinformatics and reproducible research in high-throughput biology. Annals of Applied Statistics.

[bib4] Barnhart HX, Kosinski AS, Haber MJ (2007). Assessing individual agreement. Journal of Biopharmaceutical Statistics.

[bib6] Begley CG (2013). Six red flags for suspect work. Nature.

[bib5] Begley CG, Ellis LM (2012). Raise standards for preclinical cancer research. Nature.

[bib7] Bloom T, Ganley E, Winker M (2014). Data access for the open access literature: PLOS's data policy. PLOS Biology.

[bib8] Bogardus ST, Concato J, Feinstein AR (1999). Clinical epidemiological quality in molecular genetic research - the need for methodological standards. Journal of the American Medical Association.

[bib9] Brazma A (2009). Minimum information about a microarray experiment (MIAME) - successes, failures, challenges. The Scientific World Journal.

[bib10] Chalmers I, Glasziou P (2009). Avoidable waste in the production and reporting of research evidence. Lancet.

[bib11] Collins FS, Tabak LA (2014). NIH plans to enhance reproducibility. Nature.

[bib12] Corey DR, Wise JA, Fox KR, Stoddard BL (2014). Breakthrough articles: putting science first. Nucleic Acids Research.

[bib13] Easterbrook PJ, Berlin JA, Gopalan R, Matthews DR (1991). Publication bias in clinical research. Lancet.

[bib15] Errington TM, Iorns E, Gunn W, Tan FE, Lomax J, Nosek BA (2014). An open investigation of the reproducibility of cancer biology research. eLife.

[bib16] Freedman LP, Inglese J (2014). The increasing urgency for standards in basic biologic research. Cancer Research.

[bib17] Glasziou P, Altman DG, Bossuyt P, Boutron I, Clarke M, Julious S, Michie S, Moher D, Wager E (2014). Reducing waste from incomplete or unusable reports of biomedical research. Lancet.

[bib18] Hooft L, Bossuyt PM (2011). Prospective Registration of marker evaluation studies: time to act. Clinical Chemistry.

[bib19] Hurlbert SH (1984). Pseudoreplication and the design of ecological field experiments. Ecological Monographs.

[bib20] Institute of Medicine (2012). Evolution of Translational Omics: Lessons Learned and the Path Forward.

[bib21] Ioannidis JP, Greenland S, Hlatky MA, Khoury MJ, Macleod MR, Moher D, Schulz KF, Tibshirani R (2014). Increasing value and reducing waste in research design, conduct, and analysis. Lancet.

[bib22] Ioannidis JP (2005). Why most published research findings are false. PLOS Medicine.

[bib23] Ioannidis JP (2006). Journals should publish all ‘null’ results and should sparingly publish ‘positive’ results. Cancer Epidemiology Biomarkers & Prevention.

[bib24] Ioannidis JP (2014). How to make more published research true. PLOS Medicine.

[bib25] Irizarry RA, Warren D, Spencer F, Kim IF, Biswal S, Frank BC, Gabrielson E, Garcia JG, Geoghegan J, Germino G, Griffin C, Hilmer SC, Hoffman E, Jedlicka AE, Kawasaki E, Martínez-Murillo F, Morsberger L, Lee H, Petersen D, Quackenbush J, Scott A, Wilson M, Yang Y, Ye SQ, Yu W (2005). Multiple-laboratory comparison of microarray platforms. Nature Methods.

[bib26] Lambert CG, Black LJ (2012). Learning from our GWAS mistakes: from experimental design to scientific method. Biostatistics.

[bib27] Macleod MR, Michie S, Roberts I, Dirnagl U, Chalmers I, Ioannidis JP, Al-Shahi Salman R, Chan AW, Glasziou P (2014). Biomedical research: increasing value, reducing waste. Lancet.

[bib28] Maecker HT, McCoy JP, Immunophenotyping FH (2011). A model for harmonizing flow cytometry in clinical trials. Nature Immunology.

[bib29] McNutt M (2014). Reproducibility. Science.

[bib30] Moher D, Schulz KF, Altman DG (2001). The CONSORT statement: revised recommendations for improving the quality of reports of parallel-group randomised trials. Lancet.

[bib31] Morrison SJ (2014). Time to do something about reproducibility. eLife.

[bib32] Nature (2013). Reducing our irreproducibility. Nature.

[bib33] Nosek BA, Lakens D (2014). Registered reports. Social Psychology.

[bib34] Piantadosi S (2005). Clinical Trials: A Methodological Perspective.

[bib35] Parker HS, Leek JT (2012). The practical effect of batch on genomic prediction. Statistical Applications in Genetics and Molecular Biology.

[bib36] Prinz F, Schlange T, Asadullah K (2011). Believe it or not: how much can we rely on published data on potential drug targets?. Nature Reviews Drug Discovery.

[bib37] Schulz KF, Altman DG, Moher D, CONSORT Group (2010). CONSORT 2010 statement: updated guidelines for reporting parallel group randomised trials. Journal of Clinical Epidemiology.

[bib38] Sebastiani P, Solovieff N, Puca A, Hartley SW, Melista E, Andersen S, Dworkis DA, Wilk JB, Myers RH, Steinberg MH, Montano M, Baldwin CT, Perls TT (2011). Retraction. Science.

[bib39] Stegle O, Parts L, Piipari M, Winn J, Durbin R (2012). Using probabilistic estimation of expression residuals (PEER) to obtain increased power and interpretability of gene expression analyses. Nature Protocols.

[bib14] The Economist (2013). Unreliable research: trouble at the lab. The Economist (19 October 2013).

[bib40] Westgard JO, Barry PL, Hunt MR, Groth T (1981). A multi-rule shewhart chart for quality-control in clinical-chemistry. Clinical Chemistry.

